# Supplementary material for: Adequacy of Pharmacovigilance Training in the Health Professional Training Institutions in Uganda: Training Gaps and Opportunities for Improvement
Source: Med Sci Educ. 2024 Oct 4;35(1):193–204. doi: 10.1007/s40670-024-02162-1 (PMC11933492; doi:10.1007/s40670-024-02162-1)
Supplement: Supplementary file 1 — Supplementary file1 (DOCX 89 KB) [file 40670_2024_2162_MOESM1_ESM.docx]

**Adequacy of pharmacovigilance training in the health professional training institutions in Uganda: Training gaps and opportunities for improvement.**

Rajab Kalidi^1^, Henry Kyeyune^1^, Sula Balikuna^1^, Hassan Matovu^1^, Julius Mayengo^2^ and Helen Byomire Ndagije^2*^

1. Department of pharmacy, School of Health Sciences, College of Health Sciences, Makerere University, Kampala, Uganda; [kalidi.rajab@mak.ac.ug](mailto:kalidi.rajab@mak.ac.ug) (R.K), [henry.kyeyune@mak.ac.ug](mailto:henry.kyeyune@mak.ac.ug) (H.K), [baliksh@gmail.com](mailto:baliksh@gmail.com) (S.B), [matovuhassan@yahoo.com](mailto:matovuhassan@yahoo.com) (H.M), [jmayengo@nda.or.ug](mailto:jmayengo@nda.or.ug) (J.M), [hndagije@nda.or.ug](mailto:hndagije@nda.or.ug) (H.B.N.).
2. Directorate of Product Safety, National Drug Authority, Kampala, Uganda

*Correspondence: [hndagije@nda.or.ug](mailto:hndagije@nda.or.ug)

**Abstract:**

**Background:** Despite efforts to improve Pharmacovigilance systems, Uganda’s reporting rate remains below the WHO effective reporting criteria of 200 reports per a million inhabitants annually. Adequate education of health science students on pharmacovigilance is one of the core sustainable interventions to improve pharmacovigilance systems. This study assessed the adequacy of pharmacovigilance training in health professional training institutions in Uganda in order to identify the current needs and improvement opportunities.

**Methods:** Data was collected from Allied health professional training institutions offering courses in clinical medicine and community health and diploma in Pharmacy; Universities offering Bachelor’s degrees in Medicine and Surgery, Pharmacy, Nursing and Dental Surgery, and Nursing training institutions offering certificate and diploma courses in Nursing and Midwifery. The study involved review of 16 curricula and 18 interviews with the heads of the programs of the different institutions. Data on pharmacovigilance content covered in the training curricula, challenges in pharmacovigilance training, pharmacovigilance competencies, knowledge, and skills gaps were collected. The study also included 13 key informants from policy and regulatory bodies, Pharmaceutical industry, importers and distributors of pharmaceuticals, professional councils/societies, examination boards, and Hospitals to get perspectives on training gaps and opportunities for improvement. Quantitative data was analyzed using Microsoft Excel 2017. Qualitative data were transcribed and reported verbatim.

**Results:** Most of the curricula 16(80%) had content on medication use problems though inadequate and only 1 (5%) had content on causality assessment. Majority of the respondents from the training institutions 12 (67%) reported having no staff with subject specific training background on pharmacovigilance. Other challenges reported include lack of instructional materials and time on their already packed curricular to teach pharmacovigilance. All the respondents from training institutions 18 (100%) and key informants 13 (100%) recommended incorporation of pharmacovigilance into pre-service training curricula as a means of improving pharmacovigilance training and competencies among graduates. According to the key informants, there is need to strengthen pharmacovigilance training in the pre-service curriculum 9 (100%). The knowledge and skills that should be strengthened included detection, management and causality assessment 4 (44%) and spontaneous safety reporting 3 (33%).

**Conclusions:** The curricula for health professional training institutions do not adequately cover content on pharmacovigilance. The key areas that should be strengthened are detection, management and reporting of medication use problems, and causality assessment. Pharmacovigilance content should be introduced and/ or strengthened in the existing curricula of all health professional training institutions to meet the growing need for pharmacovigilance experts, create culture of medicine safety and vigilance, and improve patient safety.

**Key points**

| The pharmacovigilance content covered in health professional training institutions in Uganda is inadequate. |
| --- |
| Pharmacovigilance content in the training curricula of health care professional training institutions should be enhanced for students to increase their vigilance in preventing and reporting of adverse drug events. |

**Running head**

Review of pharmacovigilance curricula of health professionals’ training institutions in Uganda.

**1.0 Background**

Pharmacovigilance (PV) is the science and activities relating to the detection, assessment, understanding and prevention of Adverse Drug Reactions (ADRs) or any other medicine/ vaccine related problems (WHO 2002). This important science, PV, is applied globally to prevent medicine related ADRs and improve public health and safety through assessment of benefits, effectiveness and risk of harm from medicine use (WHO 2002, WHO 2014). PV requires continuous monitoring of the safety of drugs on the market in order to detect, prevent and control ADRs that could not be identified during the clinical phases (Nadew, Beyene *et al*. 2020). PV data is generated through filling and collecting ADR reports from healthcare workers and patients at the end of the supply chain. However, under-reporting of ADRs continues to be a major global and national challenge (Varallo, Guimarães et al. 2014, NDA 2020). In Uganda the reporting rate is still below the average of adequate reporting centers (Ndagije, Nambasa et al. 2019). For example, in the second quarter of 2020, only 38.20% reports, the highest number of reports in the country were received from facilities in Kampala (NDA 2020). The low rate of reporting of ADRs underestimates the magnitude and risk related to safety and efficacy of medicines impacting decision making and potentially causing serious harm to the patient and increased health care costs (Kiguba, Karamagi *et al.* 2014, Katusiime, Semakula *et al*. 2015, Potlog Shchory, Goldstein *et al*. 2020).

The main contributing factors from studies to the under-reporting of ADRs among health care professionals include; lack of knowledge and awareness of the ADR reporting systems, inadequate teaching and training of ADR reporting in undergraduate, internship and postgraduate studies, delayed feedback from the National Pharmacovigilance Centers (NPCs), fear of legal litigation, unavailability of reporting formats and indifference of professionals (Kiguba, Karamagi et al. 2014, Varallo, Guimarães *et al*. 2014, Katusiime, Semakula *et al*. 2015, Gurmesa and Dedefo 2016, Nadew, Beyene *et al*. 2020). Therefore, addressing knowledge gaps on PV should be a major intervention to prevent medicine related ADRs and improve public health and safety. Enhancement of knowledge among health science students creates awareness on their obligations as future healthcare professionals in the PV system and benefits of spontaneous reporting of ADRs, and increases their vigilance in preventing ADRs (Shankar, Subish *et al*. 2006, Seselja Perisin, Bukic *et al*. 2021).

However, previous studies have reported inadequacies in the education curricula of health science students on PV (Shankar, Subish *et al*. 2006). More so, WHO recommends NPCs to advocate for incorporation and or strengthening of PV into the training curricula of health care professional training institutions. In addition, the Uganda National PV strategy 2019-2024 provides for the incorporation of PV into the national training curricula of health workers as a key intervention to enhance knowledge and skills of personnel at all levels of the health care system on PV. This study assessed the extent of coverage of PV content in curricula currently used for the training of different healthcare cadres. It also sought to identify challenges in PV training, knowledge and skills gaps in order to highlight opportunities for improvement.

**2.0 Methods**

**2.1 Study design, setting and population**

This was a cross-sectional study conducted from June to August 2022. The study included allied health professional training institutions offering courses in Clinical Medicine and Community Health and Diploma in Pharmacy; universities offering bachelor’s degrees in Medicine and Surgery (MBChB), Pharmacy, Nursing and Dental Surgery (BDS); and Nursing training institutions offering certificate and diploma courses in nursing and midwifery. Of the 64 allied health training institutions recognized by Allied Health Professional Council (AHPC) in 2022, 23 offer diploma courses in clinical medicine and community health and or Pharmacy (AHPC 2022). There are 87 recognized nursing training institutions by Uganda Nurses and Midwives Council (UNMC) in 2022 (UNMC 2022). These include 11 government, 27 faith based and 34 private nursing schools and 15 Universities. The accredited Universities offering medical courses in Uganda include six (6) public Universities (Busitema University, Gulu University, Soroti University, Kabale University, Makerere University and Mbarara University of Science and Technology) and five (5) private Universities (Clarke International University, Kampala International University, Uganda Martyrs University, St. Augustine International University and Islamic University In Uganda). In addition to the academic institutions, the study also included key informants (KIs) from policy and regulatory bodies, Pharmaceutical industry, importers and distributors of pharmaceuticals, professional councils/societies, examination boards, and Hospitals.

The study population included Deans/heads of department for the Universities, principals/tutors from the allied and nurses training institutions and selected KIs. The study also involved review of curricula for the different health professional training institutions.

All registered and recognized health training institutions by relevant accrediting bodies and pharmaceutical industry, distributors and importers with Qualified Person for Pharmacovigilance (QPPV) were included in the study. Hospitals which actively participate in the reporting of ADRs were also included. The other institutions were included by virtue of their role in regulation of training and practice of healthcare professionals in the country. Health professionals’ training institutions that were in existence for less than three (3) years or had not graduated students were excluded from the study.

**2.2 Sample size and sampling procedure**

*2.2.1 Training institutions:* The study targeted review of at least two curricula per program from the different institutions to determine the extent of coverage of PV content in the curricula. As shown in table 1 below, a total of 37 curricula from 10 training institutions were reviewed and interviews were held with 18 deans/heads of departments and principals/tutors. The institutions were sampled based on a combination of both convenient and purposive sampling. The institutions offering programs of interest were purposively selected while those offering more programs of interest and near each other were conveniently selected. The deans/heads of departments and principals/tutors were purposively selected based on their knowledge and influence in curriculum development to identify challenges in PV training at their institutions.

*2.2.2 Key informants*: A total of 13 KIs were purposively selected to identify challenges in PV training, knowledge and skills gaps among graduates and generate perspectives on opportunities for improvement. These included KIs from professional councils/associations (4), Uganda Allied Health Examination Board (UAHEB) (1), Uganda Nurses and Midwives Examinations Board (UNMEB) (1), NDA (1), pharmaceutical distributors and importers (2), Pharmaceutical industry (2), Hospitals (2), and MOH pharmacy Department (1).

*Table 1 showing selected institutions*

| **Programs** | **Selected Institutions** | | | | | | | | | | |
| --- | --- | --- | --- | --- | --- | --- | --- | --- | --- | --- | --- |
|  | MUK | MUST | KIU | GU | UIAHMS- Mulago | Gulu SCO | Gulu SNM | FINS | Jinja SNM | Nsambya SNM | Total |
| Bachelor of medicine and surgery | 1 | 1 | 1 | 1 |  |  |  |  |  |  | 4 |
| Bachelor of Pharmacy | 1 | 1 | 1 |  |  |  |  |  |  |  | 3 |
| Bachelor of Nursing/midwifery | 1 | 1 | 1 | 1 |  |  |  |  |  |  | 4 |
| Bachelor of Dentistry | 1 |  | 1 |  |  |  |  |  |  |  | 2 |
| Diploma in Pharmacy |  |  | 1 | 1 | 1 | 1 |  |  |  |  | 4 |
| Diploma in Clinical medicine and community health |  |  | 1 |  | 1 | 1 |  |  |  |  | 3 |
| Diploma in Nursing |  |  | 1 |  |  |  | 1 | 1 | 1 | 1 | 5 |
| Certificate in nursing |  |  | 1 |  |  |  | 1 | 1 | 1 | 1 | 5 |
| Diploma in midwifery |  |  |  |  |  |  | 1 | 1 | 1 | 1 | 4 |
| Certificate in Midwifery |  |  |  |  |  |  | 1 | 1 |  | 1 | 3 |
| **Overall total number of Programs/curricula** | | | | | | | | | | | **37** |

*Universities: MUK- Makerere University Kampala, MUST- Mbarara University of Science and Technology, GU- Gulu University and KIU- Kampala International University; Allied Health and nursing training institutions: UIAHMS- Uganda Institute of Allied Health and Management Sciences, SCO- School of Clinical Officers, SNM- School of Nursing and Midwifery and FINTS- Fortportal International Nurses training School*

**2.3 Data collection tools, methods and analysis**

A questionnaire, data abstraction checklist and an interview guide were used to collect data. The questionnaire was administered to Deans/Heads of Department and Principals of the training institutions. The data abstraction checklist was used to extract details of pharmacovigilance content in the curriculum. The interview guide was administered to the key informants. The tools were pre-tested prior to the data collection process to ensure validity and clarity of the questions. Questions found to be ambiguous were revised prior to actual data collection. The pre-test acted as part of orientation and training of research assistants. The pre-test was conducted in non-participating institutions and results of the pre-test were excluded from the final analysis. Prior to the data collection, letters and emails were written to the participants seeking for their participation in the study. Those that accepted to participate were further briefed on the day of the study about the study and oral consent sought from them before administration of the tools. The questionnaire and interviews took about 30 minutes to complete.

Data obtained were sorted, coded and entered into Microsoft Excel spreadsheet for analysis. Descriptive statistics i.e., percentage and frequency were used to summarize data obtained. Other qualitative data were transcribed and reported verbatim. Emerging quotes from the interviews were highlighted and marked for referencing.

**3.0 Study findings**

## 3.1 Respondent characteristics

The respondents from Health Training Institutions (HTIs) included mainly principals 9 (50%) and heads of department 5 (28%). The majority of the key informants were in charge of quality control 3 (23%) and registration of professionals 3 (23%) at their institutions. (Table 2)

Table 2: Respondent characteristics

| **Characteristic** | **Frequency** | **Percentage (%)** |
| --- | --- | --- |
| **Respondents from HTIs** | **n=18** |  |
| Dean | 4 | 22 |
| Head of Department/designee | 5 | 28 |
| Principal/designee | 9 | 50 |
|  |  |  |
| **Key informants** | **n=13** |  |
| In-Charge Quality Control | 3 | 23 |
| Ag. Manager Pharmacovigilance | 1 | 8 |
| Registrar | 3 | 23 |
| Vice president/chair education committee | 1 | 8 |
| Hospital Pharmacist | 2 | 15 |
| Examination officer | 2 | 15 |
| PV in-charge | 1 | 8 |

## 3.2 Pharmacovigilance course content in the curriculum

Most of the curricula 16(80%) had content on medication use problems though inadequate and only 1 (5%) had content on causality assessment**. Details in figure 1, tables 3 and 4.**

Figure 1 PV content covered in the curriculum

Table 3: Pharmacovigilance content in the curriculum (Degree courses)

| **Bachelor of pharmacy** | |
| --- | --- |
| Curriculum 1 2018 edition | - ADRs (terminologies, classification, causes of adverse effects) and drug interactions |
| Curriculum 2 2016 edition | - ADRs: types of adverse effects; side effects, toxic effects, idiosyncratic, iatrogenic, teratogenic, hypersensitivity - Drug safety, medication errors and drug interactions - Causality assessment - Introduction to pharmacovigilance, data-bases and systems used in pharmacovigilance - Drug utilization studies, evaluating and improving physician prescribing, bias and confounding - Special issues in studying vaccine safety, the drug approval process. |
| Curriculum 3 2022 edition | - Introduction to pharmacovigilance, data-bases and systems used in pharmacovigilance - Drug utilization studies, evaluating and improving physician prescribing, bias and confounding - Special issues in studying vaccine safety, the drug approval process |
| **Bachelor of Medicine and Bachelor of Surgery (MBChB)** | |
| Curriculum 1 2018 edition | - ADRs and pharmacogenetics: terminologies, classification, influence of genetic variability to response to drugs |
| Curriculum 2 2016 edition | - ADRs and pharmacogenetics: terminologies, classification, influence of genetic variability to response to drugs |
| Curriculum 3 2020 edition | - Management of medicines, prescribing-drug safety, ADR, prescribing and rational drug use |
| Curriculum 4 2022 edition | - Therapeutic Drug monitoring, ADR reporting and monitoring |
| **Bachelor of Dentistry** | |
| Curriculum 1 2019 edition | - ADRs and pharmacogenetics: terminologies, classification, influence of genetic variability to response to drugs |
| Curriculum 2 2016 edition | - ADRs and pharmacogenetics: terminologies, classification, influence of genetic variability to response to drugs |
| **Bachelor of Nursing** | |
| Curriculum 1 2018 edition | - ADRs and pharmacogenetics: terminologies, classification, influence of genetic variability to response to drugs |
| Curriculum 2 2016 edition | - ADRs and pharmacogenetics: terminologies, classification, influence of genetic variability to response to drugs |

Table 4: Pharmacovigilance content in the curriculum (Diploma and certificate courses)

| **Diploma in clinical medicine and community health** | |
| --- | --- |
| Curriculum 1 2019 edition | - ADRs and pharmacogenetics: terminologies, classification, influence of genetic variability to response to drugs |
| Curriculum 2 2016 edition (National) | - Definitions of ADRs, drug interactions and substance of abuse |
| **Diploma in Pharmacy** | |
| Curriculum 1 2016 (National) | - ADRs and Drug interactions |
| Curriculum 2 2015 | - Definition of side effect and drug interactions |
| **Diploma and Certificates in Nursing and Midwifery** | |
| National Curricula (2017, 2018 editions) | - No content |

## 3.3 Pharmacovigilance knowledge and skills gaps

The key informants who are practitioners and or knowledgeable on PV were asked regarding gaps in PV knowledge and skills. According to them, there is need to strengthen PV training in the pre-service curriculum 9 (100%) and the PV practitioners should be able to diagnose, detect, manage and report ADRs 4 (44%) and perform causality assessment 2 (22%). The knowledge and skills that should be strengthened included detection, management and causality assessment 4 (44%), spontaneous safety reporting 3 (33%), and reporting and data analysis 2 (22%). Details in table 5.

Table 5: Pharmacovigilance competencies, knowledge and skills gaps

| **Category** | **Frequency n=9** |
| --- | --- |
| **Need to strengthen PV training in the curriculum** |  |
| Yes | 9 |
| No | 0 |
| **Reasons for need of PV in the curriculum** |  |
| - Healthcare professionals as they interface with patients need to report adverse events | 4 |
| - PV is not in the curriculum, it is not formalized or curriculum has basic content covered under pharmacology | 3 |
| - It’s important to optimize patient treatment outcomes | 1 |
| - Few experts in PV yet the market is demanding more | 1 |
| **Competencies required for PV practitioners** |  |
| - Ability diagnose expected side effects/adverse drug reactions | 4 |
| - Ability to detect, manage and report ADRs | 4 |
| - Causality assessment of ADRs | 2 |
| - Capacity to collect and analyze data on ADRs | 2 |
| - Risk profiling, risk management, precautionary measures and reporting | 1 |
| - Conduct surveillance, research, drug regulation and M&E | 1 |
| **Knowledge and skills that need to be strengthened** |  |
| - Detection, management and causality assessment | 4 |
| - Spontaneous safety reporting | 3 |
| - Writing aggregate reports on how the molecule works | 2 |
| - Validation and investigation of ADRs | 2 |
| - Data analysis and reporting- for pharmacists | 2 |
| - Basics of PV, its importance and application | 2 |
| - Identification of risks and how to communicate the risks to patients | 2 |
| - Use of real time/ electronic reporting platforms and usage of existing tools | 2 |
| - Attention to labels that follow the products | 1 |
| - Knowledge on expected ADRs and ability to advise patients to always report side effects | 1 |

*“The competencies will vary based on the level and specialty. At lower levels such as diplomas, the focus should be on detecting, monitoring, documenting and reporting adverse drug reactions. At higher levels such as degree, aspects of surveillance, research, drug regulation, M&E, etc can be added to the above (KI, professional council/association)”.*

*“Most undergraduate curricula mention PV only in passing, and there is no serious focus on PV as a practice or regulatory requirement (KI, pharmaceutical importer/distributor)”.*

*“Data collection and reporting should be strengthened because often times, the quality of data captured is wanting and cannot be relied on to make meaningful decisions (KI, pharmaceutical industry)”.*

## 3.4 Teaching and incorporation of PV in the training curricula of health professional training institutions

*3.4.1 Health training institution respondents*

Majority of the respondents 12 (67%) reported having no staff with training background on PV and they recommended a standalone 10 (56%) PV course/module for incorporation into the curricula. All the respondents 18 (100%) recommended incorporation of PV in the curriculum and 7 (39%) recommended a 3 credit unit course/module.

*3.4.2 Key informant responses*

All the key informants 13 (100%) also recommended incorporation of PV in the curriculum to meet the growing need for PV experts 3 (23%), create culture of medicine safety and vigilance 3 (23%), and improve patient safety and drug efficacy 2 (15%). Majority of the key informants also recommend a standalone 9 (69%) PV course/module for incorporation into the curricula.

Table 6 shows the details.

Table 6 : Details of how to incorporate PV in the training curriculum

| **Category** | **Frequency (Percentage)** | **Frequency (Percentage)** |
| --- | --- | --- |
| **Staff trained to teach PV** | **n=18** |  |
| Yes | 6 (33) |  |
| No | 12 (67) |  |
| **PV is taught as an independent course/module** | **n=20** |  |
| Yes | 0 (0) |  |
| No | 20 (100) |  |
| **PV should be incorporated into the curriculum** | **n=18** | **n=13** |
| Yes | 18 (100) | 13 (100) |
| NO | 0 (0) | 0 (0) |
| **How PV should be incorporated** | **n=18** | **n=13** |
| Standalone module or course | 10 (56) | 9 (69) |
| Incorporated into the existing curriculum | 8 (44) | 4 (31) |
|  |  |  |
| **Number of contact hours for PV content** | **n=18** |  |
| 1 CU | 2 (11) |  |
| 3CU | 7 (39) |  |
| NOT SURE | 9 (50) |  |
| **Why PV incorporation into the pre-service curriculum** | **n=18** | **N=13** |
| - Good knowledge and skills about PV promotes medication safety and prevent medication errors | 5 (28) | 2 (15) |
| - Improve knowledge on PV so that trainees have the ability to perform PV activities | 2 (11) | 2 (15) |
| - Students need to be taught such that they prevent/detect and report ADRs | 1 (6) | 1 (8) |
| - Growing need for PV experts globally to protect the patients, who are the biggest stakeholders in the drug industry |  | 3 (23) |
| - Creates a safety culture and vigilance early in healthcare professionals so that they know from an early stage that it’s part of what they are supposed to do |  | 3 (23) |
| - It will ensure better treatment outcomes and quality of drugs |  | 2 (15) |
| - Others | 10 (56) | 3 (23) |

*Others: Creates an opportunity to formulate better molecules than those phased out because of the side effects; it is more cost-effective and sustainable, and achieves more impact since many trainees are targeted and when incorporated at an early stage (undergraduate), there are increased chances of picking interest in it at postgraduate level.*

*“ADRs can be fatal, lead to serious disability and costly to manage; students need to be taught such that they prevent/detect ADRs (HTI respondent)”.*

*“PV is required especially for public health, pharmacy and medicine, many ADRs occur but are not reported, professionals are not adept with reporting of ADRs. So it is critical for those working with patients and drugs to be deliberately trained on PV (HTI respondent)”.*

*“Incorporating PV into the pre-service curriculum will improve patient safety and drug efficacy since the health care professional will be better equipped to monitor the after-effects of the drugs and other aspects like counterfeits (KI, pharmaceutical industry)”.*

*“Integrating PV training into the undergraduate curricula is more cost-effective and achieves more impact since many trainees are targeted. It also makes horizontal integration with other relevant courses such as pharmacology, therapeutics, clinical pharmacy, and drug regulation. Pre-service training also makes PV training more easily sustainable at a national level (KI, professional council/association)”.*

*“PV should be incorporated in only clinical courses i.e. clinical medicine, public health dentistry and orthopedics but not public health and rehabilitation programs such as physiotherapy, occupational therapy, environmental health Science (KI professional council/association)”.*

## 3.5 Challenges of incorporation of PV in the training curricula of health professional training institutions and suggested solutions

The key challenges reported by both key informants and health training institutions were lack of human resource capacity to teach PV, lack of instructional materials and time to teach PV given the overloaded curricula. The main solutions suggested for successful incorporation of PV were training of trainers, examination of PV, provision of training resources for PV and sensitization of stakeholders on PV. Details in table 7 and 8.

Table 7: Challenges envisioned regarding incorporation of PV in pre-service curriculum

| **Challenges** | **Frequency (Percentage) n=13** | **Frequency (Percentage) n=18** |
| --- | --- | --- |
|  | **KI responses** | **Training institution responses** |
| - Lack of human resource capacity to teach PV | 9 (69%) | 8 (44%) |
| - Lack of instructional materials/teaching aids | 2 (15%) | 6 (33%) |
| - Adjusting the curriculum to create time for PV given the fact that there is a lot to cover under PV | 6 (46%) | 4 (22%) |
| - Ensuring that the content is tailor made to programs |  | 2 (11%) |
| - It requires time to convince the different stakeholders to change their attitude and embrace PV | 3 (23%) | 1(6%) |
| - Limited financial resources to facilitate the trainings | 1 (8%) | 1(6%) |
| - Appreciation of PV by the training institution will be a challenge because many of them may not understand its importance | 1 (8%) |  |
| - Politics and bureaucracy within the training institutions might delay or fail the incorporation of PV in the pre-service curriculum. | 1 (8%) |  |
| - The content may be ignored if not examined |  | 1(6%) |
| - Disjointed curriculum reviews |  | 1(6%) |

*“The PV course will need a trained expert to teach it which has implications on wage bill if the expert is to be hired. Moreover, getting enough trained PV experts to run these courses across all Health training Institutions (HTIs) in the country might be a challenge. The course will also need specifically designed/prepared teaching materials/resources to ensure a hands-on training (HTI respondent)”.*

*“PV is a core area in pharmaceutical care delivery except that disjointed development of curricula makes it difficult to incorporate such in some national curriculum given the irregular review schedules (HTI respondent)”.*

Table 8: Suggestions for successful incorporation of PV in pre-service curriculum

| **Suggestions for successful incorporations** | **Frequency (Percentage) n=13** | **Frequency (Percentage) n=18** |
| --- | --- | --- |
|  | **KI responses** | **Training institution responses** |
| - Training of trainers (tutors/lecturers), to support PV training | 4 (31%) | 9 (50%) |
| - Work with examination boards to make PV examinable | 2 (15%) | 5 (18%) |
| - Provide reference materials and learning resources | 1 (8%) | 3 (17%) |
| - Sensitize different stakeholders before introduction | 2 (15%) | 1 (6%) |
| - PV should be taught during the third year of study and should offer practical knowledge/skills in terms of summary of product characteristics and also avail relevant softwares | 1 (8%) |  |
| - First pilot-test PV with core programs like Pharmacy and Medicine to inform the whole process of PV training and then scale down afterwards to other programs | 1 (8%) |  |
| - Provide the incentive and motivation by making PV part of the internship package | 1 (8%) |  |
| - Proper documentation of everything required teach PV | 1 (8%) |  |
| - Involve the PV centre to initially support the training institutions as they build their capacity | 1 (8%) |  |
| - Bench mark from successful institutions | 1 (8%) |  |
| - Involve the practitioners of PV in the training | 1 (8%) | 2 (11%) |
| - Each institutions should be asked to tailor content, depth according use to their professions |  | 1 (6%) |
| - PV should be incorporated in second year or 3^rd^ year for degree courses and second semester 1^st^ year for diploma and certificate courses |  | 1 (6%) |

*“Involve the National Pharmacovigilance centre to initially support the training institutions as they build their capacity. The centre can interest its visitors who are experts in PV to support the Institutions (KI policy and regulation)”.*

# 4.0 DISCUSSION OF FINDINGS

This study assessed the extent of coverage of PV content in health professional training institutions in Uganda in order to identify the current training needs, establish the challenges to the training and generate perspectives on opportunities for improvement. While there was no curricular with a dedicated course or module on PV, the majority had cursory topics on aspects of PV taught as part of broader courses. The number and content of topics on PV on the different curricular varied with the levels of the programs. University programs particularly the Bachelors of Pharmacy had the most and broadest topics while diploma and certificate courses in Nursing had limited or no content on PV. The varied patterns of coverage of PV content in the different curricular may be due to differences in real and perceived responsibility of a particular profession in PV. While over the years the importance of PV has increasingly been recognised, some respondents at HTI believed it to be a duty principally for pharmacists and thought training on it should be more emphasised in pharmacy curricular. This perception may in part explain the differences in content of PV covered. Further, most respondents thought their curricular were already packed with little room for addition of new topics or courses and so when considerations are made during curricular review, subjects such as PV are not prioritised for they are deemed non-core to training of non-pharmacy HCPs. More so, lack of expertize or training in pharmacovigilance and national core curriculum course on pharmacovigilance might have contributed to the limited content of PV covered in the curriculum. The curricular course content for different health programs are developed by the lecturers/tutors in the training institutions and are mainly a reflection of their opinion of what is important, individual expertize, preference or personal purposes *(*Beckmann, Hagemann *et al*. 2014, van Eekeren, Rolfes *et al*. 2018). This among others calls for development of national core curriculum to guide teaching of PV in HTIs. From literature, teaching important aspects of PV to medical and health science students requires a core curriculum that describes desired competencies and learning outcomes and provides practical materials*(*van Eekeren, Rolfes *et al*. 2018). Programs at Bachelors and Diploma all contained topics on ADR and drug interactions which are core topics in the WHO-International Society of Pharmcovigilance (ISoP) curriculum on PV(Beckmann, Hagemann *et al*. 2014). This coverage is just 7 % of WHO-ISoP curriculum. No curriculum had practical training of any kind on PV content. There is therefore need to strengthen education of PV to build knowledge of and raise awareness about PV.

The PV knowledge and skills for students acquired during their education are crucial for their contribution to future safe use of medicines early in their career (van Eekeren, Rolfes *et al*. 2018). Previous literature suggests that health care students may recognize the importance of ADR reporting and express the intention to report ADRs but they are insufficiently prepared to handle ADRs and have inadequate PV competencies (Alshakka, Bahattab *et al*. 2017, Othman, Ibrahim et al. 2017, Schutte, Tichelaar *et al*. 2017). In a study identifying gaps and opportunities for improving teaching of PV, most students reported that PV was not adequately covered in the curricula (Seselja Perisin, Bukic *et al*. 2021). As reported in the same study identifying gaps and opportunities for improving teaching of PV, the inadequacies in knowledge and skills tend to be more among medical, dental and nursing students than pharmacy students (Seselja Perisin, Bukic *et al*. 2021). In this current study, in line with previous studies, all the key informants reported need to strengthen PV training in the pre-service curriculum. The knowledge and skills to be strengthened reported in this current study included detection, management, causality assessment, spontaneous safety reporting, and data analysis. The WHO PV core curriculum for university teaching also prescribes understanding the importance of pharmacovigilance and preventing, recognizing, managing, and reporting adverse drug reactions as key aspects that should be covered (Beckmann, Hagemann et al. 2014). From literature, limited PV knowledge of health care professionals is the main reason for underreporting of ADRs (Seselja Perisin, Bukic *et al*. 2021). There is therefore need to strengthen teaching of PV to increase the knowledge and awareness of PV among aspiring future healthcare professionals to improve their handling of ADRs in clinical practice and reporting them. This will promote optimal patient care and prudent use of medicines now and in the future.

The teaching and incorporation of PV in the pre-service curricula of health professional programs is critical to sustainably improve the current pharmacovigilance systems. Incorporation of PV in training curriculum ultimately improves the detection, recognition and timely reporting of medicine safety by the future health care professionals. The WHO PV core curriculum for university teaching focusses on clinical aspects and can be integrated into existing courses such as pharmacology and pharmacotherapy or used as a stand-alone course (Beckmann, Hagemann *et al*. 2014). In the current study, all respondents appreciate the importance of PV and agree that it should be incorporated into the pre-service training curricula for all aspiring health care professionals. The content as proposed by the respondents can either be introduced as a stand-alone course/module or incorporated in an existing one though most favour a standalone course/module. The arguments for a standalone module/course on PV included need to emphasis PV and giving sufficient time for teaching of PV content to allow learners to comprehend the content better. However, a standalone course may require more extensive curriculum review to find the appropriate time and credit units for the new content. Many curricula reviewed are already bulky such that any incorporation of new courses/modules becomes a balancing act that requires replacement of content deemed less core to the program. Introducing PV content as part of an existing module/course requires increasing the number of credit units for an existing course and thus may be quicker to implement. However, the content would be taught along with the already existing content and may therefore not be emphasized to the same extent as it would be in a standalone module/course, and may not be examined. In Uganda, curricula for universities are developed using the NCHE format whereas those for allied health professionals training institutions are developed using the format prescribed by BTVET. As such, different curricula should be developed following the respective formats for universities and allied health professionals training institutions. Furthermore, universities develop and revise their own curricula for the different programs independently and set their own examinations. This implies that the decision to incorporate the PV content, how and when it is done can vary from university to university. However, health professionals’ councils/associations such as PSU, UMDPC, UAHPC and UNMC and the examination bodies such as UAHEB and UNMEB influence the content in the respective curricula for the training of their cadres. All these bodies are therefore central to the adoption of any new content in the curriculum and should be engaged. The lack of staff with specific training in PV can deter successful implementation of this program. There will be need to prepare and provide training and reference materials to the facilitators in these institutions.

## Limitations

The study employed non-probability methods of sampling which might have introduced bias, and the sample may not be a representation of the entire population. This was minimized by including different types of HTIs such as private, government and private not profit and most of the key stakeholders.

# 5.0 CONCLUSIONS

The curricula for health professional training institutions generally lack content on pharmacovigilance. There is inadequate content on medication use problems but barely any content on pharmacovigilance systems and methods, reporting of medication use problems, and causality assessment and signal detection. Incorporation of pharmacovigilance in the training curriculum was recommended by all the respondents and the key challenges to incorporation of pharmacovigilance in the curricula envisioned were lack of human resource to teach pharmacovigilance, lack of instructional materials and time to teach pharmacovigilance. The main solutions suggested for successful incorporation of pharmacovigilance are training of trainers, examination of pharmacovigilance, provision of training resources for pharmacovigilance, and sensitization of stakeholders on pharmacovigilance.

## 6.0 Recommendations

1. Pharmacovigilance content should be introduced and or strengthened in the existing curricula of all health professional training institutions.
2. A national model pharmacovigilance curriculum focusing on clinical aspects should be developed for incorporation into the curriculum of all health professional training institutions. There should be two curricula developed, one for diploma and certificate programs and the other for degree programs. The pharmacy training programs can add industrial aspects of pharmacovigilance in their curriculum in addition to clinical aspects proposed as a core for all health professional programs.
3. National pre-service training manual on pharmacovigilance should be developed and trainers and assessors/examiners should be trained on pharmacovigilance using the manual to facilitate its incorporation and teaching.

# 7.0 REFERENCES

Alshakka, M., et al. (2017). "Comparison of the knowledge and perception of pharmacovigilance among pharmacy, dental and medical students in Aden-Yemen." **3**(4): 254-261.

Beckmann, J., et al. (2014). "Teaching pharmacovigilance: the WHO-ISoP core elements of a comprehensive modular curriculum." **37**(10): 743-759.

Gurmesa, L. T. and M. G. Dedefo (2016). "Factors affecting adverse drug reaction reporting of healthcare professionals and their knowledge, attitude, and practice towards ADR reporting in Nekemte Town, West Ethiopia." BioMed Research International **2016**.

Katusiime, B., et al. (2015). "Adverse drug reaction reporting among health care workers at Mulago National Referral and Teaching hospital in Uganda." Afr Health Sci **15**(4): 1308-1317.

Kiguba, R., et al. (2014). "Recognition and reporting of suspected adverse drug reactions by surveyed healthcare professionals in Uganda: key determinants." BMJ Open **4**(11): e005869.

Nadew, S. S., et al. (2020). "Adverse drug reaction reporting practice and associated factors among medical doctors in government hospitals in Addis Ababa, Ethiopia." Plos one **15**(1): e0227712.

NDA (2020). "<NDA-Annual-Pharmacovigilance-report-2019_2020-pdf.pdf>."

NDA (2020). "<Pharmacovigilance Bulletin-quarter-2-_3 (1).pdf>." **12**(4).

Ndagije, H., et al. (2019). "PO 8438 Knowledge, attitude and perceptions on adverse drug events reporting among patients and healthcare providers in rural uganda." BMJ Global Health **4**(Suppl 3): A37-A37.

Othman, G. Q., et al. (2017). "Knowledge and perception about pharmacovigilance among pharmacy students of Universities in Sana’a Yemen." **11**(6): FC09.

Potlog Shchory, M., et al. (2020). "Increasing adverse drug reaction reporting—How can we do better?" Plos one **15**(8): e0235591.

Schutte, T., et al. (2017). "Pharmacovigilance skills, knowledge and attitudes in our future doctors–A nationwide study in the Netherlands." **120**(5): 475-481.

Seselja Perisin, A., et al. (2021). "Teaching Pharmacovigilance to Healthcare Students: Identifying Gaps and Opportunities for Improvement." Pharmacy **9**(3): 147.

Seselja Perisin, A., et al. (2021). "Teaching Pharmacovigilance to Healthcare Students: Identifying Gaps and Opportunities for Improvement." **9**(3): 147.

Shankar, P. R., et al. (2006). "Teaching pharmacovigilance to medical students and doctors." Indian journal of pharmacology **38**(5): 316.

van Eekeren, R., et al. (2018). "What future healthcare professionals need to know about pharmacovigilance: introduction of the WHO PV core curriculum for university teaching with focus on clinical aspects." **41**(11): 1003-1011.

Varallo, F. R., et al. (2014). "Causes for the underreporting of adverse drug events by health professionals: a systematic review." Revista da Escola de Enfermagem da USP **48**: 739-747.

WHO (2002). Importance of pharmacovigilance-safety monitoring of medicinal products. Uppsala: The Uppsala Monitoring Centre; 2002.

WHO (2014). WHO pharmacovigilance indicators: A practical manual for the assessment of pharmacovigilance systems. Who,(1), 1–5.

**Declarations**

**Funding:** This study was funded by World Health Organization (WHO) through Makerere University School of Public Health. The publication fees for open access were provided by Uganda National Drug Authority.

**Conflicts of Interests:** The authors declare that they have no conflicts of interest.

**Availability of data and materials:** All the data for the study have been provided in the manuscript.

**Ethics approval and consent to participate**

This study involved review of documents and interviews to inform development of a national curriculum for pharmacovigilance. Ethical approval or exemption was not sought. However, permission was sought from the participating institutions before data collection. Informed consent using the School of Health Sciences Research and Ethics Committee informed consent template was obtained from the participants before they took part in the study. Confidentiality and data anonymity were ensured at all times by exclusion of direct participant and institution identifiers from the study tools.

**Consent for publication***:* Not applicable

**Code availability***:* Not applicable

**Author Contributions:** Conceptualization, K.R, J.M and H.B.W; Data curation and formal analysis, K.R, H.K, S.B and H.M; Methodology, K.R, J.M and H.B.W; Project administration, K.R, J.M and H.B.W; Supervision, K.R, J.M and H.B.W; Validation, K.R, J.M and H.B.W; Visualization, K.R, J.M and H.B.W; Writing – original draft, K.R, H.K, S.B and H.M; Writing – review and editing, K.R, H.K, S.B, H.M, J.M and H.B.W.

**Acknowledgments:** We acknowledge Makerere University School of Public Health for overall project management and coordination. We specially acknowledge the Dean School of Public Health Prof. Rhoda Wanyenza and Ms. Jackie Nanteza for the project management and coordination efforts. We also acknowledge Dr. Mwoga Joseph from WHO Uganda office for providing supervision and guidance during the implementation of the project.
